# A Single-Session Preliminary Evaluation of an Affordable BCI-Controlled Arm Exoskeleton and Motor-Proprioception Platform

**DOI:** 10.3389/fnhum.2015.00168

**Published:** 2015-03-30

**Authors:** Ahmed Mohamed Elnady, Xin Zhang, Zhen Gang Xiao, Xinyi Yong, Bubblepreet Kaur Randhawa, Lara Boyd, Carlo Menon

**Affiliations:** ^1^MENRVA Research Group, School of Engineering Science, Simon Fraser University, Burnaby, BC, Canada; ^2^Brain Behaviour Laboratory, Department of Physical Therapy, Faculty of Medicine, University of British Columbia, Vancouver, BC, Canada

**Keywords:** stroke, exoskeleton, BCI, electric stimulation, proprioception

## Abstract

Traditional, hospital-based stroke rehabilitation can be labor-intensive and expensive. Furthermore, outcomes from rehabilitation are inconsistent across individuals and recovery is hard to predict. Given these uncertainties, numerous technological approaches have been tested in an effort to improve rehabilitation outcomes and reduce the cost of stroke rehabilitation. These techniques include brain–computer interface (BCI), robotic exoskeletons, functional electrical stimulation (FES), and proprioceptive feedback. However, to the best of our knowledge, no studies have combined all these approaches into a rehabilitation platform that facilitates goal-directed motor movements. Therefore, in this paper, we combined all these technologies to test the feasibility of using a BCI-driven exoskeleton with FES (robotic training device) to facilitate motor task completion among individuals with stroke. The robotic training device operated to assist a pre-defined goal-directed motor task. Because it is hard to predict who can utilize this type of technology, we considered whether the ability to adapt skilled movements with proprioceptive feedback would predict who could learn to control a BCI-driven robotic device. To accomplish this aim, we developed a motor task that requires proprioception for completion to assess motor-proprioception ability. Next, we tested the feasibility of robotic training system in individuals with chronic stroke (*n* = 9) and found that the training device was well tolerated by all the participants. Ability on the motor-proprioception task did not predict the time to completion of the BCI-driven task. Both participants who could accurately target (*n* = 6) and those who could not (*n* = 3), were able to learn to control the BCI device, with each BCI trial lasting on average 2.47 min. Our results showed that the participants’ ability to use proprioception to control motor output did not affect their ability to use the BCI-driven exoskeleton with FES. Based on our preliminary results, we show that our robotic training device has potential for use as therapy for a broad range of individuals with stroke.

## Introduction

Nearly 30% of stroke survivors suffer from motor and somatosensory deficits (Connell et al., 2008). Rehabilitation interventions after stroke are important to reduce motor and somatosensory deficits (Cifu and Stewart, 1999). Stroke rehabilitation is unfortunately labor-intensive and expensive. In US alone, the direct cost for stroke rehabilitation is about $36.5 billion per year (Go et al., 2014).

Researchers have been working on various approaches to reduce the cost of rehabilitation while maximizing the rehabilitation outcomes. These approaches include (1) task-specific training therapy in which individuals practice goal-directed motor tasks (Smania et al., 2003; Hubbard et al., 2009; Wong et al., 2012); (2) robotic-assisted therapies that use an exoskeleton or/and a functional electrical stimulation (FES) to conserve time, human labor, and cost (Glanz et al., 1996; Looned et al., 2014); (3) brain–computer interface (BCI) to promote mental practice and user engagement (Dobkin, 2007; Graimann et al., 2007; Daly and Wolpaw, 2008); and (4) proprioceptive feedback that could enhance the motor-related components of electroencephalography (EEG) (Ramos-Murguialday et al., 2012).

In recent years, interest has grown in robotic-assisted rehabilitation (Colombo et al., 2005; Fazekas et al., 2006, 2007; Lo et al., 2010; Loureiro et al., 2011; Poli et al., 2013). Rehabilitation robotic devices aim to assist individuals with stroke who have limited mobility. However, therapies that involve passive robotic-assisted movements are not as efficient, as they do not actively engage the participants during the therapy. The best outcomes are achieved when the users are actively engaged in the task and help to initiate movement of the robotic devices (Lo and Xie, 2012; Formaggio et al., 2013). To encourage user active engagement during the rehabilitation process, BCI-driven robotic devices have been proposed (Adamovich et al., 2004; Ang et al., 2009; Grosse-Wentrup et al., 2011; Ramos-Murguialday et al., 2012; Webb et al., 2012). A BCI system allows the user to translate his/her intention to commands that operate a device such as a computer and/or a robotic arm (Wolpaw et al., 2002). In order to activate a BCI-driven robotic device, the user performs motor imagery or mental practice of motor tasks. Studies have suggested that the combination of exercises and mental practice with BCI helps in neurological recovery perhaps by driving both anatomical and functional reorganization within the central nervous system (Di Pino et al., 2014).

Growing evidence, from both human and animal models, have shown that learning brings about a transformation in the way brain process information (Elbert et al., 1975; Lebedev et al., 2000). These plastic changes are needed to consolidate and store acquired sensorimotor knowledge. Therefore, enhancing the brain plasticity by maximizing the use of remaining undamaged brain, can improve functional recovery following brain injury (Chen et al., 2002; Wang et al., 2010). Further, combining mental practice with robotic motion and feedback can help to augment the sensorimotor feedback to influence functional ability in individuals with stroke (Broetz et al., 2010; Caria et al., 2011; Karolyn, 2011). Interestingly, in a recent study by Ramos-Murguialday et al. (2012), the proprioceptive feedback provided by a BCI-controlled exoskeleton enhanced the motor-related signals of the brain of healthy individuals.

No studies have combined BCI, robotic exoskeletons, FES, and proprioceptive feedback into a rehabilitation platform that facilitates goal-directed motor movements. The primary objective of this paper was to combine all these technologies and test the feasibility the device. We have developed a BCI-driven exoskeleton with FES that can assist individuals with stroke in performing goal-directed motor tasks. This device is lightweight and portable. Besides, it provides proprioceptive feedback similar to Ramos-Murguialday et al. (2012) study. More specifically, the users see and feel their hand with the exoskeleton moving through when working through the goal-directed motor task. We are also interested in understanding whether the motor-proprioception ability of stroke individuals affects their ability to use the BCI-driven device. Thus, a motor task that requires proprioception for completion was developed to assess motor-proprioception ability.

## Materials and Methods

This section first provides details about the three main components of the BCI training device: an exoskeleton arm, a FES unit, and the BCI system. Next, it elaborates how these components are integrated and used to assist the users in performing goal-directed motor tasks. Finally, we present the experimental procedures used in this study. All of the methods within this study were in compliance with the declaration of Helsinki (Bosnjak, 2001) and were approved by the Simon Fraser University (SFU) Office of Research Ethics (# 2012s0527).

### BCI training device

The BCI training device we developed operated to assist stroke individuals in performing a pre-defined goal-directed motor task. This device consists of (1) a lightweight and portable exoskeleton arm, (2) a FES unit, and (3) a BCI. The exoskeleton provides assistance to perform elbow extension and flexion. The FES unit assists in performing finger flexion and extension, which enables grasping or releasing an object. Finally, the BCI allows the users to drive both the exoskeleton arm and the FES unit by motor imagery.

#### Exoskeleton arm

**Figure 1:**
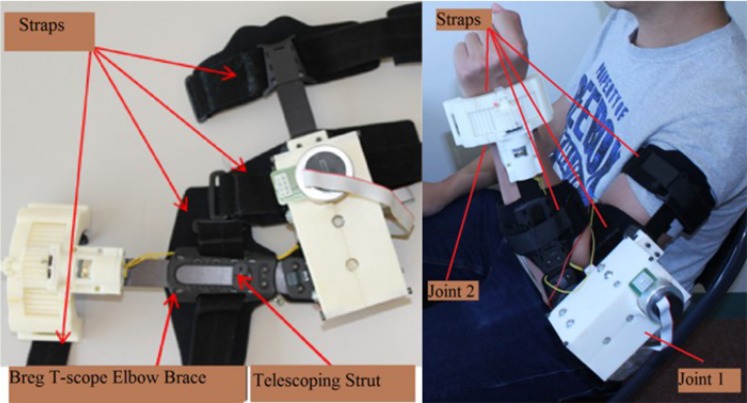
**Exoskeleton prototype**. The exoskeleton is composed of three parts: the metallic structure, the elbow joint (Joint 1), and the forearm joint (Joint 2). The metallic structure is an off-the-shelf Breg T-Scope Elbow brace, which has telescoping struts to allow maximum customization of fit. The Breg T-Scope Elbow brace also provides an extension and flexion control from 10° to 120°. Moreover, for safety purposes, it has an adjustable mechanical lockout. Besides, the Breg T-Scope Elbow brace has four straps: two for the forearm and two for the upper arm. These straps ensure full attachment between the brace and the user’s arm.

Figure 1 depicts the prototype of the exoskeleton arm developed in our lab. The exoskeleton arm has two degrees of freedom (DOF), i.e., elbow flexion/extension and forearm pronation/supination. Each DOF can be controlled independently. The elbow flexion/extension and forearm pronation/supination are actuated via “Joint 1” and “Joint 2,” respectively. “Joint 1” is actuated via a brushless DC (BLDC) motor and a customized gearbox. The BLDC motor provides a torque of 69.5 mNn at a nominal speed of 3480-rpm at 79% max efficiency. The customized gearbox consists of a casing and six reduction stages. The casing is rapid prototyped from acrylonitrile butadiene styrene (ABS) plastic. The six reduction stages are made of off-the-shelf plastic spur gears and each stage has a reduction ratio of 1:3. The angular displacement of “Joint 1” is measured via a 256–2048 CPT, 2 channels, with line drive Maxon MILE Encoder. Theoretically, “Joint 1” is able to provide 40 Nm torque. However, for safety purposes, we limited the output torque to 10 Nm. The angular speed is also limited to 2 /s.

“Joint 2” is actuated via a brushed DC planetary gear motor and a single-stage customized gearbox. The brushed DC geared motor provides a torque of 1247 mNm at a nominal speed of 38.7 rpm at 24.11% efficiency. The customized single-stage gearbox has a ratio of 1:3.5 and is rapid prototyped from ABS plastic. The angular displacement of the wrist pronation/supination is measured via a low profile and long life EVWAE Panasonic potentiometer. Theoretically, the wrist joint is able to provide 4.36 Nm torque at a nominal speed of 11 rpm. However, for safety purposes, we limited the output torque to 2 Nm. The angular speed was, on the hand, limited to 15°/s. The range of motion (ROM) was limited to 45° for wrist extension and 45° for wrist flexion with the use of a mechanical stop.

There are several advantages of the exoskeleton. First, the exoskeleton is lightweight (total weight approximately 1 kg). Its’ actuator is driven by three 12 V batteries and thus, the exoskeleton is portable. Second, the user can wear the exoskeleton in <30 s when aided by another person. Both of the exoskeleton’s joints can be positioned in such a way that the exoskeleton does not to interfere with the user’s natural arm position when he/she is relaxed or performing tasks. These features are desirable and enhance the usability of the exoskeleton when assisting the users in performing goal-directed movements in daily activities and exercises for rehabilitation purposes.

#### Functional electrical stimulation

The FES unit used in this study is the RehaStim I (Hasomed GmbH, Magdeburg, Germany), which has eight stimulation channels. RehaStim I has the capacity to generate biphasic rectangle pulses with a frequency range of 1–140 Hz, a pulse width range of 20–500 μs, and a current output range of 0–130 mA.

#### Brain–computer interface

The BCI used in this study is a non-invasive and EEG-based system. EEG signals are acquired from the users using an inexpensive wireless EEG system, i.e., Emotiv EPOC neuroheadset (Emotiv SDK Research Edition Specifications, 2010). The sampling rate of the EEG signals is 128 Hz. The headset consists of 14 channels: AF3, F7, F3, FC5, T7, P7, O1, O2, P8, T8, FC6, F4, F8, and AF4; with reference electrodes at the P3/P4 locations.

The EEG signals acquired from the headset were first processed by a signal processing unit that extract useful features the signals. Then, the features were translated by a classifier to control signals that were used to activate the exoskeleton and the FES unit. The BCI discriminated two classes of EEG signals, i.e., rest and motor imagery. Thus, the output of the classifier had one of the two discrete states “0” (rest) or “1” (motor imagery). The logical states “1” indicated the user’s intention to activate the device (the exoskeleton arm or the FES in this case). The logical states “0,” on the other hand, implied that the user did not intend to activate the device.

#### System integration and control

**Figure 2:**
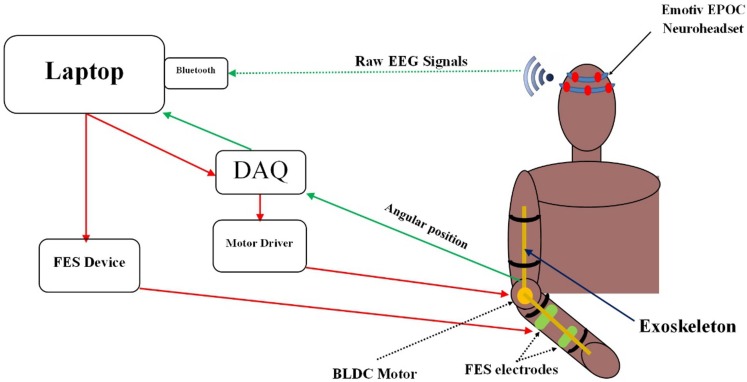
**Functional components of the training system**. EEG data were collected and processed by the BCI software embedded in the laptop. Control signals were then generated by the BCI to trigger the exoskeleton or the FES unit.

Figure 2 illustrates the functional components of the BCI training device with system software running on a laptop. The BCI system was integrated with the FES unit and the exoskeleton using LabVIEW. First, the EEG data of a user were transmitted from the Emotiv headset to the laptop via Bluetooth. Then, the EEG data were processed using the BCILAB software. The BCI generated an output every 0.5 s. As per established two-class classification, if the BCI output was a “0,” nothing happened. If the BCI output was a “1,” a control signal was sent to: the motor driver through a NI USB-6341 Data Acquisition System (DAQ) to trigger the exoskeleton or the FES unit to assist hand opening. Meanwhile, the angular position of the exoskeleton was continuously sent to the laptop via the DAQ. This data served as a feedback signal to move the exoskeleton to the desired position.

**Figure 3:**
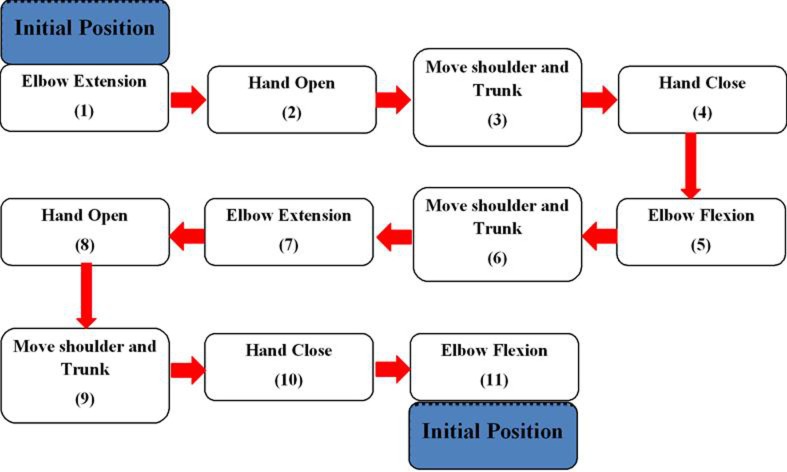
**Flowchart of the goal-directed motor task**. The task required one to move a cup from an initial position to a new position. It was divided into 11 phases (movements). All of these phases are triggered by the positive outputs of the BCI, except Phase 3, 6, and 9.

The training device operated in a preprogramed movement sequence to assist its users in performing a goal-directed motor task. The task was divided into 11 phases (Figure 3). Each phase involved movements of the shoulder, the elbow, or the hand. Depending on which phase the participant was at, the BCI would trigger (1) the exoskeleton to assist elbow extension/flexion; (2) the FES unit to assist hand opening/closing; or (3) no device for phases that involve shoulder/trunk movements. More details of the different phases of the exercise are provided in Section “Training Protocol.”

### Experimental protocol

To evaluate the performance of the BCI training device, nine stroke participants were recruited. During the training session of the experiment, the participants went through BCI training (30–45 min) and the FES unit set up (10–20 min). Next, they used the training device to perform goal-directed motor tasks (15–30 min). The participants were also assessed for their motor-proprioception ability before and after the training session using a motor-proprioception assessment protocol we developed.

#### Participants

Participants with chronic stroke (>6 months post) were recruited through local rehabilitation hospitals and stroke clubs. All potential participants were screened to meet the following inclusion criteria: (a) age range from 35 to 85 years, (b) post-stroke duration ≥6 months, (c) Montreal Cognitive Assessment (MoCA) ≥25 (Aggarwal and Kean, 2010), or pass any other cognitive assessment test, (d) shoulder active ROM in all directions of 10°–15°, (e) elbow passive extension and flexion ROM of 0°–130°, (f) wrist passive extension ROM of 0°–15°, and (g) fingers full passive extension. The exclusion criteria included (a) any other neurological conditions in addition to stroke, (b) unstable cardiovascular disease, (c) contraindications to FES, or (d) other conditions (e.g., poor sitting balance) that precluded them from undergoing the study.

Further, we used the upper-extremity subtest of the Fugl-Meyer (FM) test to examine the impairment severity of all stroke participants (Gladstone et al., 2002). We also used kinesthetic and visual imagery questionnaire short version (KVIQ-10) (Malouin et al., 2007) to quantify the motor imagery ability of the participants.

Nine male stroke participants (mean age 66 ± 11.9 years) agreed to participate in this study. The demographics and pre-assessment results of the participants are presented in Table 1. One participant (P01) had expressive aphasia [15/30 on the Frenchay Aphasia Assessment (Al-Khawaja et al., 1996)] and was unable to complete the MoCA test. Nevertheless, he was still included in this study to explore if individuals with expressive aphasia could operate the training device. The FM scores of the participants ranged from 11 to 63, suggesting mild to severe motor impairments. All participants were also tested on the KVIQ to assess their imagery ability. All participants scored above 29 out of 50.

**Table 1:** **Stroke participants’ demographics and pre-assessment data**.

Participant ID	Age	DAS (months)	HH	AA	MOCA	FM	KVIQ
P01	64	102	R	R	N/A	11	29
P02	60	99	R	L	30	31	43
P03	69	18	R	L	26	13	35
P04	67	37	R	L	25	27	43
P05	72	43	L	L	25	57	32
P06	38	18	R	L	26	31	40
P07	78	14	R	L	25	30	35
P08	66	42	R	L	26	63	37
P09	82	36	R	L	26	22	36
M&SD	66.2 ± 11.6	45.4 ± 33	–	–	–	–	–

*DAS, duration after stroke; HH, handedness; AA, affected arm; FM, Fugl-Meyer score; KVIQ, kinesthetic and visual imagery questionnaire score; M&SD, mean and standard deviation*.

#### Training protocol

All the stroke participants learn how to use the training device to perform a pre-defined goal-directed motor task. The training protocol involved three consecutive steps as explained below: the setup of the BCI and the FES unit, and the use of the BCI-driven device to perform the goal-directed motor task.

##### BCI setup

The Emotiv headset was applied to the participant’s head. Each participant was seated comfortably in front of a laptop. To set up a BCI model, the participant had to first go through a procedure called stimulus presentation. During stimulus presentation, the participant was asked to perform different repetitive imagery tasks according to the stimulus or visual cues displayed on the laptop. Two different visual cues were presented to the participant in this study: rest and imagery grasp movements with the affected arm. Each trial lasted from 9 to 11 s, i.e., the participant was asked to perform each designated task for 5 s, followed by 4–6 s of rest. The stimulus presentation stage consisted of two sessions and each session lasted 7 min. Each session consisted of 20 trials for each task. Throughout the experiment, the participant could take a break whenever needed.

Next, the two classes of EEG data collected from the stimulus presentation procedure (i.e., rest and motor imagery) were processed to generate a customized BCI model for each participant using BCILAB (Delorme et al., 2011). To generate a BCI model, a feature extraction algorithm was first applied to extract relevant features from the EEG data. Then, a classifier was trained to discriminate the two classes of EEG data. In this study, we used the common spatial pattern (CSP) algorithm (Ramoser et al., 2000; Blankertz et al., 2008) to extract features from the EEG signals. For each electrode, three features were generated by the CSP algorithm. Meanwhile, linear discriminant analysis (LDA) was used as a classifier (Christopher, 2006). To evaluate the performance of the BCI, the 10 × 10 cross-validation method was employed (Christopher, 2006). More specifically, the data set was randomized and divided into 10-folds. Nine of the folds were used to set up the classifier and the remaining onefold was used to test the classifier. This procedure was repeated for ten times. Then, the average BCI cross-validation classification accuracy was computed.

For online testing, the BCI classified the EEG features as “0” or “1” and delivered a decision output every 0.5 s. This decision output was used to trigger the exoskeleton arm or the FES unit. An output with a logical state “1” would trigger the next movement of the training device. An output with a logical state “0,” on the other hand, would not trigger anything.

##### FES setup

The RehaStim FES unit was used in the training device to assist the participants in grasping and releasing an object. For each participant, two self-adhesive rectangular electrodes were attached to the stroke affected extensor digitorum. The stimulus amplitude and the electrode positions were carefully adjusted until hand opening was achieved. Whenever the FES unit was triggered, symmetrical biphasic pulses, with a fixed frequency of 35 Hz and a peak duration of 150 μs (Bigland-Ritchie et al., 1979) were applied to open the hands of the participants. This helped the participants to adjust hand position to get ready to grasp a cup. Then, the FES unit was deactivated to release hand extension and grasp the object.

##### Goal-directed motor task

After setting up the BCI and the FES unit, the investigators helped the participant to wear the exoskeleton. The exoskeleton was secured to the participant’s arm with four straps that were fastened distally and proximally to the participant forearm and lower arm (Figure 1).

Next, an explanation on how to use the training device to perform a pre-defined goal-directed motor task was given to each participant. This task required the participants to move a cup from an initial position to a new position. This exercise was divided into 11 phases as shown in Figure 3. The participants placed the hand at an initial position where the elbow was totally flexed. At Phase 1, the participants performed motor imagery to activate the BCI and consequently trigger the exoskeleton to extend the elbow. Then, the participant would activate the BCI to trigger the FES unit to open the hand in Phase 2. At Phase 3, the participants moved the shoulder and trunk to bring the hand close to a cup on the table. Once the hand position was adjusted, the participant would use BCI control to deactivate the FES unit in Phase 3. The deactivation of the FES unit would assist the participants to close the hand and grasp the cup. At Phase 5, to lift up the cup, the participants used the BCI-controlled exoskeleton to flex the elbow. At Phase 6 and 7, the participants moved the cup to a new position using shoulder and trunk movements and the assistance from the BCI-controlled exoskeleton (elbow extension), respectively. To release and place the cup on the table, the FES unit was triggered by motor imagery (Phase 8). Finally, to return to the initial position, the participants moved the hand away from the cup using shoulder and trunk movements (Phase 9), performed motor imagery to deactivate the FES unit to close the hand (Phase 10), and performed motor imagery again to flex the elbow (Phase 11). Note that all the phases involved BCI control except shoulder and trunk movements, as the participants had active control to move their shoulder and trunk. Each participant was trained to repeat the task multiple times without break until he/she reported muscle or mental fatigue.

#### Motor-proprioception assessment protocol

The ability of the stroke participants to use proprioceptive information to update movements was assessed twice during the experiment: once before setting up the FES unit (pre-assessment) and once after the robotic-assisted cup exercises (post-assessment). The duration between the pre- and post-assessment tests was approximately 1 h.

**Figure 4:**
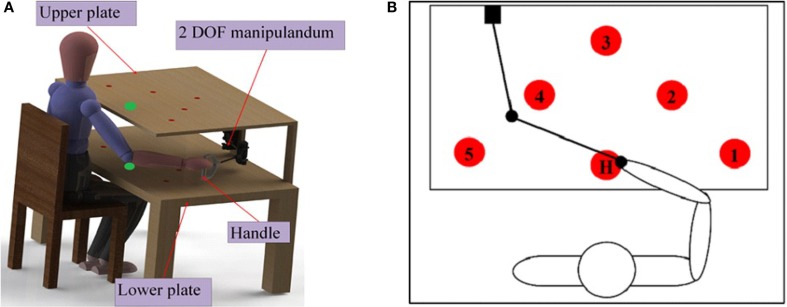
**(A)** Test bench setup for motor-proprioception assessment. The test bench has a table with two identical plates (upper and lower). The size of each plate is 50 cm × 70 cm. Each plate contains six identical circles with a diameter of 2 cm. The circles on the two plates are coaxial. **(B)** Target positions for the motor-proprioception assessment. Five of the circles on the plate (denoted by the numbers 1–5) are the target positions used for motor-proprioception assessment. H is the initial or home position.

To assess the motor-proprioception ability of the participants, we have developed a protocol using the setup shown in Figure 4. The setup consists of a test bench and a two-DOF passive manipulandum with a handle. Two potentiometers were embedded on the manipulandum handle to measure its position. The circles on the test bench are the initial (Home) and target positions the participants should aim to reach for.

During the assessment protocol, the participant’s vision of the hand, elbow, and shoulder under investigation was occluded with a drape. The participant was requested to move the handle such that the hand was placed on any of the five target positions specified by the investigator. Then, the distance error (DE) between the target position and the position achieved by the participant was calculated and recorded.

Five different sequences of target positions were used in the assessment protocol. These sequences are presented in Table 2. Each sequence contained 10 instructions that specified where the participant should reach. In each sequence, the participant was asked to reach a target position according to vocal instructions from the investigators. In these sequences, the letter “H” means that participant must return to the home position “H” and then advance to the next target position according to the vocal instruction. All sequences start from “H.”

**Table 2:** **Motor-proprioception task sequences**.

Vocal instruction steps	Sequence No.				
	1	2	3	4	5
1	1, H	1	3	4	1, H
2	2, H	2	4	1	2, H
3	3, H	3	2	3	3, H
4	4, H	4	3	5	4, H
5	5, H	5, H	5	2	5, H
6	5, H	5	3	4	5, H
7	4, H	4	1	1	4, H
8	3, H	3	3	5	3, H
9	2, H	2	4	2	2, H
10	1, H	1	2	1	1, H

*H, home target point*.

#### Motor-proprioception assessment: validity and reproducibility

To evaluate the validity of the developed method, we compared the obtained results with the test scores obtained from two other standard clinical tests for proprioception, i.e., the “up-or-down” test and the tactile sensation test. The FM test protocol for the “up-or-down” test has been used clinically to evaluate the upper-limb proprioception of a patient (Trinh, 2005). The tactile sensation test, on the other hand, was done to assesses the upper-limb tactile sensation and motor deficits quantitatively (Trinh, 2005; Dros et al., 2009). Both the “up-or-down” test and the tactile sensation test were administered to all the participants by physical therapist.

Ten healthy and eight stroke participants (all in Table 2 except P08) agreed to participate in this part of the study. All participants gave informed written consent to participate in the study. The 10 healthy participants consisted of 8 males and 2 females (aged from 22 to 37, with a mean of 27.9 ± 5.5 years; 9 right-handed, 1 left-handed).

To assess the reproducibility of the results obtained using the developed motor-proprioception assessment method, four healthy participants were asked to perform the assessment protocol twice. Two tests were conducted 7 days apart.

#### Subjective evaluation (questionnaire feedback)

At the end of the experiment, the participants were asked to complete a questionnaire to evaluate their perceived workload of using the BCI-controlled system to perform the goal-directed motor task. The questionnaire used was the NASA “task load index” (TLX) questionnaire (Hart, 2006). This questionnaire is a multifaceted assessment tool that rates perceived workload with six subscales, i.e., mental demand, physical demand, temporal demand, performance, effort, and frustration. Each participant was asked to evaluate the TLX subscales with a scale of 0–20.

## Results

### Motor-proprioception assessment test: Validity and reproducibility

#### Validity

The validity of the developed motor-proprioception assessment method was examined using the correlation values between the DE obtained from this method and the scores of two other tests (Leibowitz et al., 2008). The results are presented in Table 3. The strong correlation values between DE and the test scores from either of the standard clinical proprioception test suggest that the developed method is valid.

**Table 3:** **Validation results for the developed motor-proprioception assessment test**.

	DE vs. “up-or-down” test scores	DE vs. tactile sensation test scores
Correlation value	−0.764 (*p* < 0.001)	0.763 (*p* < 0.001)

#### Test–retest reliability

Two tests were conducted among four healthy participants, 7 days apart. For the dominant hand, the deviations (in millimeters) between the two tests’ results for each participant were 0.8, 2.8, 6.7, and 0.8, respectively (mean = 2.87 ± 2.85 mm). For the non-dominant hand, the deviations for each participant are 3.9, 5.9, 2.2, and 0.75, respectively, for non-dominant arm (mean = 3.25 ± 2.25 mm).

To quantify the reliability of the motor-proprioception assessment method, the intra-class correlation coefficient (ICC) between the data from the first test and the second test is calculated. A high intra-class correlation of 0.900 (*p* < 0.05) was demonstrated. This supports the reliability of the method.

### Training exercise results

Table 4 presents the BCI cross-validation accuracy for each stroke participant. On average, the cross-validation accuracy achieved is 68.8%, with a standard deviation of 9.0%. The results obtained from this study are similar to those reported in other BCI studies in the literature (Ang et al., 2011).

**Table 4:** **BCI cross-validation accuracy**.

Participant ID	P01	P02	P03	P05	P06	P07	P08	P09	P10
Accuracy (%)	81.10	65.20	83.20	69.26	62.87	64.25	55.78	63.31	73.90
Mean ± SD	68.76 ± 9.03								

All the stroke participants (*n* = 9) completed the training exercise at least two times. The average number of training exercise that was completed is 3 ± 0.7 (ranged from 2 to 5). The time taken to complete a trial, *T*_c_ was used to evaluate the performance of the participants. On average, the participants completed one exercise in 7.4 ± 2.8 min. All the participants successfully reduced the *T*_c_ value of the last trial (with an average of 39.3 ± 16.81 s). For three out of the nine participants (P03, P04, and P06), the reduction was <2 s. For the remaining participants, the reduction ranged from 4.9 to 128.8 s. The time to complete a trial was reduced significantly in some participants (e.g., 128.8 in P05) because after a few trials, they quickly became familiarized with the different phases of the protocol and the BCI system. Next, the difference between the *T*_c_ of the first and last trial as well as the difference between the BCI positive output of the first and last trials were also examined. No correlation was found (*r* = −0.12, *p* > 0.5) as they had gained a better control of the training device.

**Figure 5:**
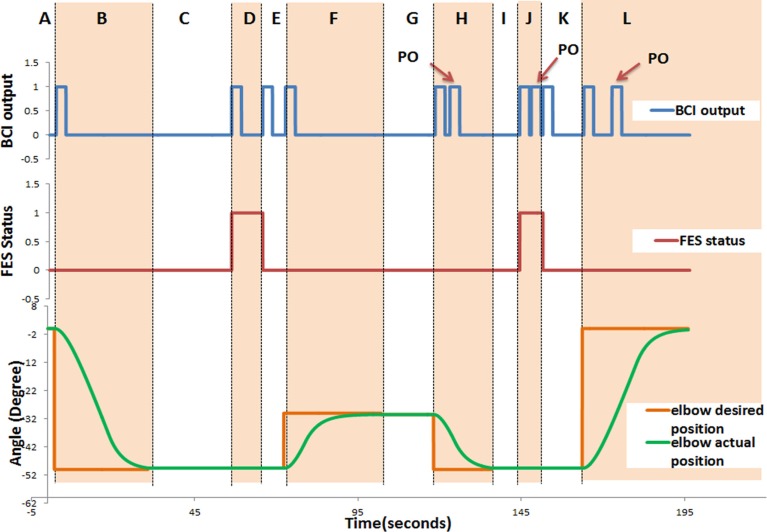
**Time course of the system response for P05**. The following data were recorded in real-time: the BCI output: rest (“0”) or activate (“1”); the FES status: deactivate (“0”) or activate (“1”); and the angular position of the exoskeleton. This figure demonstrates a typical behavior of the system. It consists of 12 regions denoted by letters from “A” to “L.” Section “A” describes the start of the trial till triggering the elbow extension, which initiates elbow extension from the initial position; Section “B” (Phase 1) describes elbow extension (to reach the cup); In Section “C,” the participant generates the trigger to activate the FES (to open the hand, Phase 2) and adjusts the shoulder/trunk (Phase 3); In Section “D,” the participant is supposed to generate a trigger to deactivate the FES (to close the hand and grasp, Phase 4); In Section “E,” the participant is supposed to generate a trigger to initiate the elbow flexion; Section “F” elbow flexion (to elevate the cup, Phase 5); In Section “G,” the participant is supposed to generate a trigger for elbow extension and adjusts the shoulder/trunk (Phase 6); Section “H” elbow extension (to place down the cup, Phase 7); In Section “I” the participant is supposed to generate a trigger to activate the FES (to open the hand and leave the cup, Phase 8); Section “J” describes the section in which the participant moves her/his shoulder/trunk away from the cup (Phase 9), and is supposed to generate a trigger to deactivate the FES (to close the hand, Phase 10); in Section “K” the participant is supposed to generate a trigger to initiate the elbow flexion; and Section “L” elbow flexion (to return to initial position, Phase 11).

For each trial of the training exercise, the following data were recorded in real-time: the BCI output: rest (“0”) or activate (“1”); the FES status: deactivate (“0”) or activate (“1”); the angular position of the exoskeleton; and the time taken to complete a trial (*T*_c_). In Figure 5, an example of the time course of the BCI output [rest (“0”) or activate (“1”)], the FES status, and the angular position of the exoskeleton for participant P05 is presented. This example shows a typical behavior of the system output in real-time when the participant was in good control of the training device. As shown in Figure 5, the participant successfully generated positive BCI outputs (“1”) that triggered the exoskeleton and the FES. However, not all positive BCI outputs in the figure are true positives. Three of the positive BCI outputs labeled as “PO” did not trigger any device. These “PO” occurred at 1.52, 0.55, and 5.65 s, respectively, after a true positive. The first and the last POs were very likely false positives because they took place more than 1 s after a true positive. These undesired positive outputs, however, did not affect the overall system performance. This is because when the exoskeleton was in operation, no other devices could be triggered.

### Motor-proprioception results of stroke participants

The motor-proprioception of the stroke participants before (pre-assessment) and after (post-assessment) the BCI training protocol were compared. Motor-proprioception was assessed using the DE values obtained from the motor-proprioception assessment protocol.

For the pre-assessment test, the DE values for the affected and non-affected arms ranged from 24.9 to 146.2 mm (mean = 60.93 ± 39.5) and 24.9 to 88.2 mm (mean = 42.3 ± 11.6), respectively. For the post-assessment test, the DE values the range for the affected arm was 28.4–124.2 mm (mean = 65.4 ± 31.13) and the range for the non-affected arm was 31.8–61.5 mm (mean = 45.47 ± 11.4). Table 5 reports the absolute difference between the DE values for the affected and non-affected arm (DDE). As the data were non-Gaussian, the Wilcoxon signed rank test was used to determine if there was any difference between the DDE values for the pre and post-assessments. The analysis suggested that there was no significant difference (*p* = 0.36).

**Table 5:** **Difference between both arms’ DE**.

Participant ID	DDE (mm)	
	Pre-assessment	Post-assessment
P01	31.6	1.2
P02	27	34.5
P03	29.2	37.1
P04	6	4.4
P05	6.3	0.5
P06	8.6	18.8
P07	16.7	8.8
P08	111.8	82.4
P09	39.7	9.2
Mean ± SD	30.8 ± 32.7	21.9 ± 26.4

The Wilcoxon signed rank test was also used to determine if there was any difference between the DDE values for the healthy and stroke participants. The analysis showed that the DDE values for the healthy and the pre-assessment DDE values for the stroke participants were statistically significant (*p* < 0.05). Interestingly, the DDE values for the healthy and the post-assessment DDE values for the stroke participants were not statistically different (*p* = 0.13). The pre-assessment DDE values for three of the stroke participants (P05, P06, and P07) were within the 95% confidence interval of the DDE values for the healthy participants, i.e., 5.5 ± 4.8 mm.

### Questionnaire feedback

At the end of the experiment, all the stroke participants subjectively evaluated the training device using the NASA TLX questionnaire. The factors taken into consideration in the questionnaire and the average ratings obtained from the participants are presented in Table 6. The results show that the mental demand is the main contributor to the workload as it has the highest average rating (14.4 ± 4.7).

**Table 6:** **NASA TLX questionnaire’s results**.

Participant ID	TLX scale (1–21)					
	Mental demand	Physical demand	Temporal demand	Performance	Effort	Frustration
P01	20	6	5	2	11	6
P02	11	5	4	0	11	0
P03	6	5	4	0	15	11
P04	17	14	16	0	15	13
P05	17	7	6	11	17	10
P06	9	3	11	3	5	0
P07	15	11	15	9	9	10
P08	19	6	12	9	13	6
P09	16	6	9	14	10	10
Mean ± SD	14.4 ± 4.7	7.0 ± 3.4	9.1 ± 4.6	5.3 ± 5.1	11.8 ± 3.7	7.3 ± 4.7

### Correlation between variables

Based on the data collected from the stroke participants, the bivariate correlations between 15 different variables were examined. This exploratory analysis allowed us to discover the relationships between these variables, which could be useful in generating hypothesis for future work. These variables include:

(1) age of the stroke individuals
(2) duration of stroke (months)
(3) upper-extremity FM scores
(4) KVIQ/KI/VI
(5) BCI cross-validation accuracy
(6) time taken to complete a trial, *T*_c_
(7) motor-proprioception ability (pre-assessment), denoted as *DDEpre*
(8) motor-proprioception ability (post-assessment), denoted as *DDEpost*
(9) difference between *DDEpost* and *DDEpre*, denoted as *DDEdif*
(10) mental demand (from TLX)
(11) physical demand (from TLX)
(12) temporal demand (from TLX)
(13) participants’ perceived performance (from TLX)
(14) effort (from TLX)
(15) frustration (from TLX).

**Figure 6:**
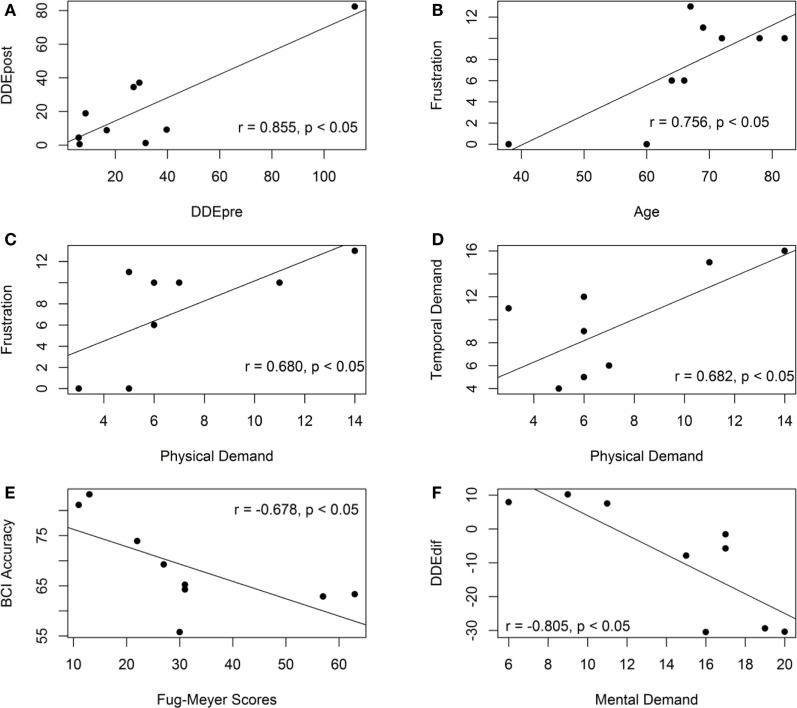
**Scatter plots of the variable pairs that demonstrate significant correlation values**. **(A)** The scatter plot of the motor-proprioception ability of the stroke participants before and after the assessment. **(B)** The scatter plot of the frustration level and age of the stroke participants. **(C)** The scatter plot of the frustration level and mental demand of the stroke participants. **(D)** The scatter plot of the temporal and physical demand of the stroke participants. **(E)** The scatter plot of the BCI accuracy and the Fugl-meyer scores. **(F)** The scatter plot of the motor-proprioception ability and mental demand of the stroke participants.

Six of the variable pairs have demonstrated significant correlation values (*p* < 0.05), i.e., motor-proprioception ability before (*DDEpre*) and after *(DDEpost*) assessment (*r* = 0.855); age and frustration (*r* = 0.756); physical demand and frustration (*r* = 0.680); temporal and physical demand (*r* = 0.682); BCI cross-validation accuracy and FM scores (*r* = −0.678); *DDEdif* and mental demand (*r* = −0.805). The scatter plots of these variable pairs are presented in Figure 6.

The motor-proprioception ability of the stroke participants before and after the assessment was highly correlated (*r* = 0.855) as demonstrated in Figure 6A. Their ICC value was 0.910 (*p* < 0.05), which also demonstrated the reproducibility of the results of the developed motor-proprioception assessment method.

The correlation analysis showed that the participant’s age and duration of stroke did not have a significant correlation with either the BCI cross-validation accuracy or the time taken to complete a robotic-assisted exercise, *T*_c_. The age of the participants, however, was correlated with their frustration level when operating the BCI-driven device (see Figure 6B). Another variable that had a positive correlation with frustration was physical demand (*r* = 0.680) as shown in Figure 6C. On the other hand, mental demand was not correlated with frustration. More specifically, those who considered the BCI-driven exercise mentally demanding (P01 and P08) did not feel more discouraged and frustrated.

As shown in Figure 6E, the BCI cross-validation accuracy had a negative correlation with the upper-extremity FM scores (*r* = −0.678). This contradicts another study in the literature (Ang et al., 2008), where the authors showed that the upper-extremity FM scores of the hemiparetic stroke patients (*n* = 35) was not linearly correlated with the BCI performance and thus, motor impairment did not affect the stroke patients’ ability to operate a BCI.

Next, our results showed no correlation between the FM scores and the KVIQ/KI/VI scores. This suggested that motor impairment did not affect the stroke participants’ ability to perform motor imagery. We also found that the BCI cross-validation accuracy was not correlated with KVIQ, KI, or VI (*p* > 0.2). This was consistent with the results reported in (Zich et al., 2015), in which no correlations were found between the KVIQ/KI/VI and the BCI accuracy. However, the results were not consistent with (Vuckovic and Osuagwu, 2013), where the authors reported a correlation between KI and BCI accuracy (*r* = 0.458) and VI and BCI accuracy (*r* = 0.728). In this study, the KVIQ scores were not able to predict the BCI accuracy possibly because the motor imagery task used to operate the BCI was different from the KVIQ motor imagery tasks. Further studies are necessary to investigate the relationship between BCI accuracies and KVIQ scores.

It was also interesting to investigate the relationship between the participants’ proprioceptive function and their feedback/performance during the online exercise protocol. The stroke participants were categorized into three groups according to the difference between their DDE values pre- and post-assessment:

(a) Group A (P01, P07, P08, and P09): decreased DDE values
(b) Group B (P04, P05, and P06): DDE values are in the range of those of the healthy participants
(c) Group C (P02 and P03): increased DDE values.

We found strong correlation between the change in the DDE values and the mental effort in the questionnaire (*r* = −0.801, *p* < 0.05). Group A reported that the training exercise was mentally demanding but not physically demanding. Group C reported that the training exercise was neither mentally nor physically demanding. For Group B, the participants (P05 and P06) who reported that the training exercise was mentally demanding experienced a decrease in the DDE values. On the contrary, participant P07 reported that the training exercise was not mentally demanding and showed an increase in the DDE values.

**Figure 7:**
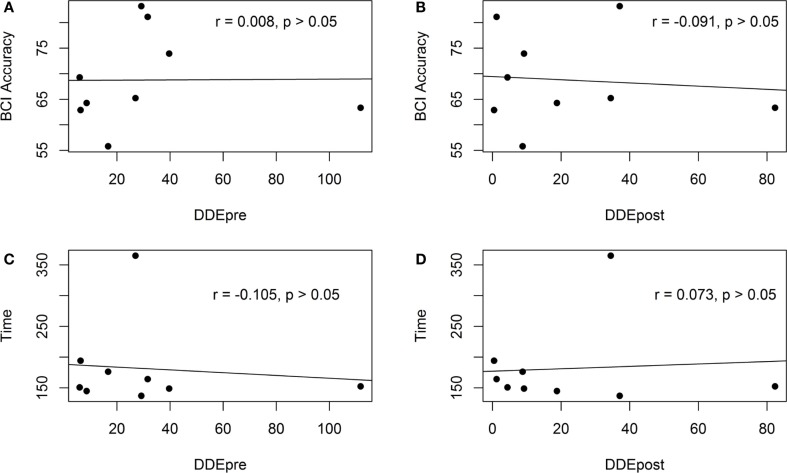
**Scatter plots of the pre- and post-assessment motor-proprioception assessment results of the stroke participants and the BCI cross-validation accuracy and the time to complete a trial**. **(A)** The scatter plot of the BCI accuracy and the motor-proprioception ability (pre-assessment). **(B)** The scatter plot of the BCI accuracy and the motor-proprioception ability (post-assessment). **(C)** The scatter plot of the time taken to complete a trial and the motor-proprioception ability (pre-assessment). **(D)** The scatter plot of the time taken to complete a trial and the motor-proprioception ability (post-assessment).

Figure 7 shows the scatter plot of the motor-proprioception ability and the BCI cross-validation accuracy/*T*_c_. The correlation analysis showed that the participants’ motor-proprioception ability did not have a significant relationship with either the BCI cross-validation accuracy or the time taken to complete a robotic-assisted exercise, *T*_c_. Our preliminary results suggested that the motor-proprioception ability of the stroke individuals and their age and duration of stroke did not affect their ability to use the BCI-driven exoskeleton with FES.

## Discussion and Conclusion

In this study, we developed a comprehensive BCI platform that combines different rehabilitation and technological approaches. The platform consists of a BCI training device and a motor-proprioception assessment protocol. The BCI training device promotes user engagement via the use of motor imagery to trigger the exoskeleton or FES. It also promotes the use of goal-directed motor task as part of the training protocol. The motor-proprioception assessment protocol provided us with a means to assess whether motor-proprioception ability was a critical factor in determining for whom this therapy may be best employed.

### Training device

To evaluate the performance of the training device, two performance metrics were examined: the BCI cross-validation accuracy and the time taken to complete a goal-directed motor task. In addition, the participants also provided subjective feedback about the system using the TLX questionnaire.

The BCI cross-validation accuracy is an important metrics widely used to assess the performance of the BCI model generated offline. Our results show that the BCI cross-validation accuracy achieved is in the range of 55.78–83.20%, which is consistent with those in the literature (Ang et al., 2011). The accuracy for some of the participants (P02, P04, P05, P06, P07, and P08) was not satisfactory (<70%). One reason is that some participants were not able to focus when performing motor imagery possibly due to their age and stroke related problems. Also, the number of electrodes placed around the motor cortex area was sparse.

For the goal-directed motor task of moving a cup, the BCI online accuracy was not available. This is because during this real-time operation, no cues were given to the participants as to when they should perform a motor imagery or rest. In other words, the system was self-paced and the participants had the flexibility of deciding when to activate the BCI and consequently trigger the exoskeleton or the FES unit. As a result, no true labels of the EEG data were available. To measure the training device performance, the time taken by the participants to complete a trial (*T*_c_) was used. On average, the participants were able to complete three trials of the exercise. The number of trials completed was small in this study because the participants experienced fatigue toward the end of the experiment. In real rehabilitation settings, the BCI set up and the use of the BCI device for training could be done on separate days. This will subsequently increase the number of trials the participants can complete. The speed of completing a trial varied across the stroke participants. The *T*_c_ values ranged from 2.3 to 6.1 min and the mean was 2.5 min. This mean value is actually quite close to that achieved when the system is ideal, i.e., approximately 2.2 min. Here, an ideal system implies that the BCI system is perfect, i.e., its accuracy is 100%. In addition, we observed that all the participants successfully reduced the *T*_c_ value of the last trial. This improvement possibly indicates that they had gained a better control of the training device. The results are encouraging. Also, our results suggest the participant with expressive aphasia was able to use the system. This demonstrates the potential use of the developed system in performing goal-directed motor task multiple times in a 1 h rehabilitation intervention.

One major factor that could affect the online performance of the system is the BCI accuracy. If the participants have a good BCI control, less time is needed to finish an exercise. To improve the performance of the BCI system, denser electrodes could be placed over the scalp especially the motor cortex area. Also, EEG signals have a low signal-to-noise ratio and are frequently contaminated with artifacts. These unwanted noises either originate from the user (e.g., ocular and facial muscle activities) or from other non-physiological sources such as power line interference and created artifacts in the EEG signal. As artifacts could affect the quality of the EEG signals and subsequently degrade the BCI performance, it is important to apply automatic artifact removal algorithms to improve the BCI performance. Another design factor that has not received much attention from BCI researchers is volitional inhibition, which is the ability to suppress a movement due to unexpected changes in the environments (Mirabella, 2012). Neural correlates of volitional inhibition have been investigated in Mirabella et al. (2011). However, more research effort is needed to decode volitional control using EEG signals before this feature is incorporated into the BCI system. The ability of the BCI to predict volitional control may potentially lead to a BCI-controlled exoskeleton/FES that better mimics natural movements (Mirabella, 2012).

### Motor-proprioception assessment

We employed our new assessment method to identify stroke participants with motor-proprioception deficits. We have demonstrated its validity by comparing its results with two other standard clinical tests for proprioception (i.e., the “up-or-down” test and the tactile sensation test). The claim is supported by the results that the DE values of the developed method has a significant correlation with the test scores obtained from either the “up-or-down” test or the tactile sensation test.

Our results show that participants P05, P06, and P07 have DDE values that were in the range of the DDE values of the healthy participants. This finding is consistent with the results obtained from the two standard clinical tests for proprioception. In addition, no correlation was found between the proprioceptive function of both the hands of the healthy participants. Thus, by comparing the proprioception of the affected and non-affected arms of the stroke participants using DDE, we can quantify the degree of which stroke has affected the proprioceptive function of the affected arm. The difference between the DDE values for the stroke participants for the pre- and post-assessments is not statistically different (*p* < 0.16). However, the average DDE values were lower after the BCI training session.

### Subjective evaluation

The stroke participants’ subjective feedback on TLX questionnaire has suggested that the developed system and exercise protocol is safe, neither slow nor fast, and not overly physical demanding. Even though the participants perceive the task as mentally demanding, they gave low frustration rating about the experiment. Based on our personal communication with the participants, their ability to control the exoskeleton and the FES unit with motor imagery has boosted their confidence level in regaining the motor functions in the future.

## Conclusion

In summary, we have developed a comprehensive platform that consists of (1) a BCI-controlled exoskeleton/FES training device with proprioceptive feedback and (2) a motor-proprioception assessment test. Despite of the small sample size in this study (*n* = 9) and the limited number of training repetitions, we showed that individuals with stroke can operate the BCI-controlled robotic training device. All the stroke participants successfully completed multiple trials of the goal-directed motor task of moving a cup using the training device. It would be interesting to investigate the use of this comprehensive platform in stroke rehabilitation at various sites of service with a bigger sample size. In our future work, a clinical study will be conducted to evaluate the efficacy of the developed platform in restoring the motor and proprioceptive functions of stroke individuals.

## Conflict of Interest Statement

The authors declare that the research was conducted in the absence of any commercial or financial relationships that could be construed as a potential conflict of interest.
